# Improving patient-centered mental health promotion in primary care in vulnerable communities through mindfulness training in Rio de Janeiro, Brazil

**DOI:** 10.3389/fmed.2024.1356040

**Published:** 2024-06-25

**Authors:** Débora Silva Teixeira, Sandra Fortes, Celia Kestenberg, Kali Alves, Mônica Rodrigues Campos, Alfredo Oliveira Neto, Francisco Ortega, Javier García-Campayo, Marcelo Demarzo

**Affiliations:** ^1^Department of Integral Family and Community Medicine, Medical Sciences College, State University of Rio de Janeiro, Rio de Janeiro, Brazil; ^2^Medical Sciences College, State University of Rio de Janeiro, Rio de Janeiro, Brazil; ^3^Nursing School, State University of Rio de Janeiro, Rio de Janeiro, Brazil; ^4^Pedro Ernesto University Hospital – State University of Rio de Janeiro, Rio de Janeiro, Brazil; ^5^National School of Public Health – Oswaldo Cruz Foundation, Rio de Janeiro, Brazil; ^6^Primary Health Care Department, Faculty of Medicine, Federal University of Rio de Janeiro, Rio de Janeiro, Brazil; ^7^Catalan Institution of Research and Advanced Study, Barcelona, Spain; ^8^Medical Anthropology Research Center, Universitat Rovira i Virgili, Tarragona, Spain; ^9^University of Zaragoza, Zaragoza, Spain; ^10^Mente Aberta – The Brazilian Center for Mindfulness and Health Promotion – Department of Preventive Medicine, Paulista Medical School – Federal University of São Paulo, São Paulo, Brazil

**Keywords:** mindfulness, public health, primary care, mental health promotion, vulnerable populations

## Abstract

**Introduction:**

Brazilian Primary Health Care (PHC) is responsible for all-sanitary actions for a community-based population, including health promotion and mental health care. Mindfulness Based Health Promotion (MBHP) is an intervention that can promote self-care and psychosocial support in PHC.

**Objective:**

To discuss the effects of mindfulness based psychosocial group interventions for health promotion in primary care units in Rio de Janeiro, Brazil.

**Methods:**

The intervention was based on the MBHP model adapted for SUS. Nine groups were held in Rio de Janeiro. A quali-quanti research was held with two parts: (a) quantitative study, pre and after the 8 weeks intervention, evaluating the effect on mindfulness and self-compassion and their association with levels of anxiety, depression, and quality of life. (b) Qualitative research using Focus Groups with the participants to investigate their experience at the end of the mindfulness groups.

**Results and discussion:**

Sixty-two participants finished the 9 groups where 86% were women, mostly between 30 and 59 years of age and low income, and around 80% under regular medical care in PHC in SUS. In the studied sample 80% had at least one chronic health condition under treatment, including 42% with anxiety and 35% with depression. The effects included significant improvement in Anxiety and Depression and in Quality of Life, mainly in the psychological but also in the physical and interrelation domains. The qualitative study showed that most patients joined the group on the recommendation of health professionals for managing physical and mental health symptoms. Patients reported being able to use the practices taught in the sessions to manage symptoms such as insomnia and emotionally distressing situations in their daily lives. Including family members in mindfulness practices was a strategy to negotiate not only a space at home to meditate, but also to obtain a different approach to health problems. Participants pointed to mindfulness as a complementary therapeutic option to medication and psychotherapy.

**Conclusion:**

Mindfulness-Based Intervention have shown to be a feasible, well-accepted and efficacious method of offering psychosocial support and promoting well-being for low-income patients in primary care in LAMIC.

## 1 Introduction

Common mental disorders (CMD), including anxious-depressive syndromes, are present in more than 50% of the population attended by Primary Health Care (PHC) units in Brazil (1), especially being associated with vulnerable social economic conditions such as violence, unemployment, and extreme poverty. CMD are frequently presented through medically unexplained symptoms, especially pain symptoms, and related to chronic diseases such as hypertension, obesity, and diabetes. They are directly associated with low self-esteem and disempowerment, indirectly associated with a strong social support network, and significantly reduce quality of life. The historical absence of psychosocial mental health interventions in PHC in the Brazilian National Health System (SUS-Sistema Único de Saúde) have led to long-lasting inadequate treatment based mainly in the prescription of benzodiazepines and the presence of important levels of treatment gap for mental disorders, such as more than 75% for depression (2). Given this situation, initiatives for scalable and cost-effective interventions are being developed in the SUS to reduce the chronic burden of CMD, promote empowerment, self-esteem, well-being and peer support.

The SUS is the largest universal public health system in the world and provides direct free health care for approximately 100% of the Brazilian population, estimated at 203 million people, although 30% also have private health insurance (3). However, SUS faces many challenges as Brazil is one of the most unequal countries in the world (4). PHC at SUS is provided by Family Health Teams (FHT), encompassing a physician, a nurse, a nurse technician and up to 6 community health workers that are responsible for all-sanitary actions for a community-based population up to 4,500 people, involving health promotion, preventive actions and solving 80% of health problems, including mental health. These teams work through a model of collaborative care work defined as matrix support (Matriciamento, or matrix support, is the Brazilian term for mental health collaborative care (MHCC) in primary health care), with other professionals such as psychologist, psychiatrist, social worker, physiotherapist, and physical educators, in an interdisciplinary work process. They are responsible for providing self-care and psychosocial support in communities cared for by the FHT, such as Community Therapy, Peer Support Groups, Handcraft Women’s’ groups, Music Therapy Group and Mindfulness Based Health Promotion (MBHP) interventions.

Since the pandemic of COVID-19 emotional distress has increased, demanding provision of primary mental health care, including financing and training of health professionals, with an extra focus on the mental health condition of the professionals (5). Given that Brazil was one of the countries hardest hit by COVID-19 in terms of the number of deaths, increased levels of anxiety and depression symptoms and a significant decrease in quality of life was observed (6). Even before the pandemic, the number of years with disability (YLDs) due to mental disorders accounted for the largest number of years of healthy life lost in Brazil resulting in 4.9 million YLDs across all age groups and corresponding to 18.8% of all YLDs in the country. Anxiety and depressive disorders represent almost one third of all non-fatal disease-related burden, which is a common scenario in low-income and middle-income countries (LAMICs) (7). The Brazilian prevalence of CMD varies from 17 to 50%, being more common in women, the elderly, people living in social isolation and at greater social vulnerability and people with other chronic health conditions (8). In the SUS, CMD cases should be managed at primary care. Due to the structural vulnerability and poverty presented by the population attending at the PHC, CMD rates among them are even higher than in the general Brazilian population: reaching up to 60% of patients in these units (9).

It is necessary, therefore, to offer non-pharmacological treatments, not only psychotherapy and supervised physical activities, but also group interventions as previously presented. The development of group interventions in PHC has been a promising strategy in the care and promotion of mental health, as it develops “safe spaces” for social inclusion and empowerment, expanding social support networks and increasing the patient’s ability to cope with adversity. Among these interventions, mindfulness has proven to be effective in PHC worldwide, both with users and health professionals experiencing burnout (10–12). In Brazilian PHC, this cost-effective intervention can offer empowerment and increase in self-esteem, as well as support from a peer group can help promote wellbeing and reduce the chronic burden of common mental disorders (13, 14).

This article examines an experience of implementation of mindfulness groups, designed especially for SUS, in PHC units in Rio de Janeiro, Brazil. Our aims are to discuss the health effects perceived by patients when participating in these mindfulness groups, identifying the challenges and facilities presented by their implementation regarding beliefs, values, expressions, experiences, and religiosity.

## 2 Materials and methods

### 2.1 Type of study

This research was designed to evaluate the effects of developing mindfulness-based interventions in primary care settings, in a “quasi experimental study (15, 16).” The first aim of the research was to train mental health and primary health care professionals in order to study the usefulness of a psychosocial group intervention based in Mindfulness Practices as part of non-pharmacological care.

To enrich our understanding of the intervention’s multifaceted impact, we employed a convergent parallel mixed-methods design, pre-post evaluation study, on the effects of the implementation of a mindfulness-based program for patients in PHC in Brazil. The research was carried out in two parts: (a) quantitative study, assessing variables pre and post the 8-week intervention with patients, (b) qualitative research using focus groups evaluating the patients experience with the program. The integration of these two parts was achieved by juxtaposing quantitative outcomes with qualitative insights (experiences and perceptions), facilitating a holistic interpretation of the intervention’s efficacy.

### 2.2 Intervention

The intervention was based on the MBHP Program designed by the Mente Aberta (Open Mind) team of Federal University of São Paulo (Unifesp) together with the State University of Rio de Janeiro (UERJ) team. It is a secular and evidence-based mindfulness training protocol, created and adopted by the “Mente Aberta” Center – Mindfulness Brazil in 2011, and has been improved over time by its general and clinical use, and by the contribution of several collaborators (17–19). The MBHP is inspired by Jon Kabat-Zinn’s original model – “Mindfulness Based Stress Reduction” (MBSR) but adapted to the context of Health Promotion and Quality of Life. In addition to MBSR, the United Kingdom Breathworks Institute, the MBCT (“Mindfulness-Based Cognitive Therapy”) and the MBRP (“Mindfulness-Based Relapse Prevention”) are the main sources of inspiration for the MBHP protocol. The program was created and designed for the Brazilian and Latin-American context, and, in particular, for its application in public policies in the areas of health, education and organizations, having as principles accessibility, didactics and simplicity, and as an objective the secular and scientific development of metacognitive awareness, providing a more conscious and healthy life, and promoting autonomy and self-care in health (17).

The groups were held at five PHC units in the city of Rio de Janeiro from 2015 till 2018, being facilitated by health professionals working in primary care units, either from the Family Health Teams or from Matrix Support Teams, under the supervision of researchers from Unifesp and UERJ. The instructors were professionals working in these units that participated in the 18 months specific training described above to work with the MBHP protocol. The groups took place in weekly meetings lasting an average of 1 h and a half to 2 h, in selected spaces in each PHC unit, organized by the instructors. The population taking part in the groups was the usual PHC clientele, referred to by the FHT that considered these patient’s needs and who could benefit from a support group intervention. Patients with severe mental health disorders such as psychotic disorders, Dementia and suicidal ideas and plans were recommended not to participate in the groups.

The patients who took part in the groups were tested with standardized instruments before and immediately after the 8-week MBHP Protocol course.

The following instruments were used in the quantitative evaluation:

### 2.3 Quantitative instruments

#### 2.3.1 Socio-demographic questionnaire and information on health status

The questionnaire aims to assess subjects’ socio-demographic data, including gender, age, if the person is only attended in the SUS or have private insurance (which is a measure of better economic status), but also asking about the existence of a chronic illness, treatments that are being currently used, including use of medication.

#### 2.3.2 Hospital Anxiety and Depression Scale

The Hospital Anxiety and Depression Scale (HADS) was developed to detect depression and anxiety in hospital environments. However, it has been shown that it has the same psychometric properties when used with the general population, especially in PHC (20). The HADS scale contains 14 questions and is subdivided into two subscales: one for anxiety and another for depression. Each subscale has seven (7) questions, the answers to which range from 0 to 3. The cutoff score of 8/9 was considered for anxiety and depression.

#### 2.3.3 World Health Organization abbreviated quality of life instrument brief

The instrument was constructed by the World Health Organization Quality of Life Group (WHOQOL Group), of the World Health Organization (WHO), for this purpose and validated in Portuguese (21). It measures, through 26 questions, quality of life in four domains - physical, psychological, social relationships and environment. The physical domain assesses pain and discomfort, energy and fatigue, sleep and rest; the psychological domain, positive feelings, thinking, learning, memory and concentration, self-esteem, body image, negative feelings; the social relations domain inquiries about personal relationships, social support and sexual activity; while the environment domain asks about the physical safety and security, home environment, financial resources, health and social care, opportunity to acquire information and skills, opportunities for recreation and leisure, physical environment and locomotion. The WHOQOL - abbreviated questions were formulated to a Likert-type response scale, with a scale ranging from 1 to 5 in intensity (nothing - extremely), capacity (nothing - completely), frequency (never - always) and assessment (very dissatisfied - very satisfied; very bad - very good).

#### 2.3.4 Full attention and consciousness scale (MAAS)

The Mindfulness and Attention Scale (MAAS) is one of the most popular scales for Mindfulness The scale allows the measurement of the construct “being attentive” based on a 15-item self-administered questionnaire to be answered with a Likert scale that varies between 1 (almost always) and 6 (almost never) and which evaluates cognitive, emotional, physical, interpersonal, and general domains (22).

#### 2.3.5 Self compassion

The Self-Compassion Scale, developed by Kristin Neff, is a psychological concept assessment tool that involves treating oneself with humility, kindness and understanding, in the same way one would treat a close friend when faced with difficulties, failures or suffering. The scale is made up of several statements that explore different aspects of self-compassion and its underlying components (23). The scale is structured around the three main components of self-compassion: self-judgment vs. self-kindness; social isolation vs. notion of shared humanity, and over-identification vs. mindfulness. Studies report positive evidence in the adaptation, validation and reliability of the use of the Self-Compassion Scale in the Brazilian population (24, 25). It has 26 items, quantifiable from “almost never” (1) to “almost always” (5).

### 2.4 Qualitative approach

Nine focus groups were held with patients by pairs of trained researchers, in the roles of moderator and observer, using a script created especially for this research. The script included questions about the participants’ expectations of the groups and their experience during the process, as well as asking about their impressions of the implementation of mindfulness-based interventions in PHC. The focus groups took place at the end of the mindfulness courses in each health unit. The groups were audio-recorded and later transcribed.

### 2.5 Data analyses

Our study involved a triangulation of methods including quantitative and qualitative methodologies. We integrated these data streams by mapping qualitative themes onto quantitative results, thereby gaining a nuanced understanding of how and why the intervention was effective. This methodological synergy allowed for a comprehensive understanding of patient outcomes beyond what could be gleaned from numerical data alone.

In the statistical analysis of the data, the paired t-test was applied to check whether there was a statistically significant difference, at the 5% level, in the scores studied between the patients’ responses before and after the intervention for all the outcomes to be assessed, namely:

- Four domains of the quality-of-life scale (WHOQOL - domains: physical, psychological, social relations and environment).
- Screening scales for anxiety (HAD-Anxiety) and screening for depression (HAD-Depression) were evaluated in terms of percentage of positive screening and on a continuous scale via the average of the sum of the scores on the scale items.
- Mindfulness and Awareness Scale (MAAS) assessed by the sum of the scores on the scale items.
- Self-Compassion Scale (SC) assessed by the average of the scores on the scale items.

In addition, this evaluation of the effectiveness of the intervention via the paired t-test was also replicated according to each of the sociodemographic and health variables investigated in the study, in order to better characterize the groups where this occurred differently.

Statistical tests were performed using SPSS Statistical software version 22 (IBM).

The qualitative material from the focus groups was analyzed based on the transcripts and the researchers’ notes, aiming for a thematic analysis. Thematic content analysis is a research method that seeks the subjective interpretation of text content through systematic classification and the identification of themes and patterns. The words used, meanings attributed by the group and ideas that emerged from the group were taken into account. The script of questions from the focus groups acted as a framework that also supported this analysis. The transcripts were read independently by the researchers in order to immerse themselves in the material. The themes and patterns identified by each researcher were listed and then discussed as a group, looking for similarities and discrepancies. Based on the identification of themes, they were categorized and the categories that emerged will be discussed below (26).

### 2.6 Procedures

The groups were facilitated by health professionals working in PHC units, under the supervision of university teams. The groups took place in weekly meetings lasting an average of one hour and thirty to two hours, in selected spaces in each PHC unit, organized by the instructors.

Nine groups were held with PHC patients in the city of Rio de Janeiro. The patients who took part in the groups were tested with standardized instruments before and after the 8-week MBHP Protocol course. A total of 144 patients started the groups, but only 62 patients completed both questionnaires (pre and post), allowing an assessment of the immediate effect of the practices implemented in the groups.

## 3 Results

### 3.1 Quantitative results

Nine groups were held with PHC patients in the city of Rio de Janeiro. A total of 144 patients started the groups, but only 62 patients completed both questionnaires (pre and post), allowing an assessment of the immediate effect of the practices implemented in the groups. 85.5% were women, mostly between 30 and 59 years of age (56.4%) and around 77% under regular medical care in PHC. The cohort analyzed (pre and post) consisted mainly of patients from middle or low middle class, with a majority without private health insurance (83.5%) indicating low income (in Brazil 73.7% of the population, mainly middle and low income classes, do not have private health insurance) (27), and were treated only in the Unified Health System. Most of them (77%) had at least one chronic health condition under treatment, including 41.9% with anxiety and 35.5% with depression, although 50% referred to suffering from depression. Meaning that 22.6% of them are not undergoing regular treatment. Although many were being treated (“taking medication”) for chronic health conditions such as hypertension, diabetes and obesity, anxiety and depression represent the second and third most frequent chronic conditions. Also 50% of patients use medication daily. We will discuss the issue of depression and anxiety below. As already described in the methodology, we evaluated the impact of mindfulness interventions especially concerning the development of mindfulness and self-compassion, but also its effects on quality of life and in the presence and intensity of depression and anxiety.

As shown in Table 1, regarding mindfulness and self-compassion skills, the interventions had a more significant effect on self-compassion. There was a significant increase in mindfulness among women with depression who, despite improving, persisted with significant depressive symptoms. However, when we evaluated the effects in terms of self-compassion, we see significant improvements for women, adults up to the age of 60, especially those with lower incomes, without chronic illness (the presence of chronic illness only indicates a tendency to improve - *p* = 0.077), and in those who receive regular monitoring for chronic illnesses including anxiety and depression. In these patients the impact of the practices was felt both in those undergoing treatment and also in those without treatment. It is important to remember that the increase in self-compassion accompanied all levels of improvement in symptoms of both anxiety and depression, even when the mental disorder was not considered to be cured.

**Table 1:** Evaluation of the mindful attention and awareness scale (MAAS), self-compassion, screening for anxiety and depression, before and after intervention, in patients of the city of Rio de Janeiro/RJ, Brazil.

Questions	Before and after	MAAS			Self-compassion		
		Before	After	Paired *t*-teste	Before	After	Paired *t*-teste
	(*n*)	(Sum)	(Sum)	*p*-value	(Mean)	(Mean)	*p*-value
All patients	**62**	**52.7**	**56.3**	**0.031**	**2.8**	**3.1**	**0.000**
Gender (Male)	9	59.9	61.2	0.772	3.1	3.2	0.385
(Female)	53	51.5	55.4	**0.028**	2.7	3.0	**0.000**
Age groups (18–29 years)	6	45.2	49.5	0.353	2.5	3.1	**0.017**
(30–59 years)	35	50.3	53.9	0.107	2.7	3.0	**0.003**
(60+ years)	17	61.2	62.6	0.644	3.0	3.1	0.245
**Do you have health insurance?**							
Yes	11	51.0	57.9	0.060	2.6	3.0	0.064
No	51	53.0	55.9	0.124	2.8	3.1	**0.000**
**Do you have a chronic illness?**							
Yes	30	54.6	55.7	0.640	2.8	2.9	0.072
No	27	51.2	55.5	0.069	2.9	3.2	**0.000**
**Do you receive regular medical care for the treatment of any ongoing or chronic health problem?**							
Yes	47	51.3	55.0	0.067	2.7	3.0	**0.000**
No	14	56.4	61.1	0.060	3.1	3.2	0.067
**Do you receive regular medical care for the treatment of anxiety?**							
Yes	26	48.8	54.0	0.057	2.6	3.1	**0.000**
No	36	55.5	57.9	0.249	3.0	3.1	0.108
**Do you receive regular medical care for the treatment of depression?**							
Yes	22	47.3	53.0	0.074	2.5	3.0	**0.000**
No	40	55.7	58.1	0.208	3.0	3.1	**0.037**
**Do you have depression?**							
Yes	29	46.8	52.7	**0.019**	2.5	3.0	**0.000**
No	29	56.6	59.0	0.321	3.0	3.1	0.268
**Do you take medication daily?**							
Yes	48	52.5	56.2	0.060	2.7	3.0	**0.000**
No	12	51.1	53.7	0.467	2.9	3.2	0.064
**Screening for anxiety**							
Negative before and after	14	66.4	68.9	0.397	3.3	3.3	0.876
Improved after intervention	11	52.8	60.3	0.059	3.0	3.4	**0.009**
Positive before and after	37	47.5	50.3	0.220	2.5	2.9	**0.000**
**Screening for depression**							
Negative before and after	19	61.5	65.6	0.177	3.3	3.3	0.797
Improved after intervention	12	56.6	59.2	0.613	2.8	3.3	**0.010**
Positive before and after	28	44.7	48.9	**0.054**	2.4	2.8	**0.000**

In red bold *p*-value < 5%.

It is important to remember that studies have shown that in primary health care the predominant condition is an association of symptoms of anxiety and depression, described as anxious depression in the ICD-11-AP proposal (28). In this way, these symptoms were evaluated together, and it was found that the effect of the intervention was positive. There was a significant reduction in the intensity of the conditions in both men and women in terms of anxiety and in women, those under 60, and those on lower incomes also in terms of the intensity of depression. There was a significant positive effect for those with depression, whether they were being treated or not. But the improvement was more significant among those who were not being treated for anxiety, regardless of whether they had chronic illnesses or not. In general, there was an association with an improvement in anxiety in general, including those associated with the presence of depression, which also reduced in intensity, even if it did not disappear completely (Table 2).

**Table 2:** Screening for anxiety (HAD-Anx) and depression (HAD-Dep), before and after intervention, in patients of the city of Rio de Janeiro/RJ, Brazil.

Questions	Before and after	HAD-Anx (categorical % positive cases)			HAD-Dep (categorical % positive cases)		
		Before	After	Paired *t*-teste	Before	After	Paired *t*-teste
	(*n*)	(%)	(%)	*p*-value	(%)	(%)	*p*-value
All patients	62	**0.77**	**0.60**	**0.001**	**0.65**	**0.50**	**0.019**
Gender (Male)	9	0.56	0.56	1.000	0.56	0.56	1.000
(Female)	53	0.81	0.60	**0.001**	0.66	0.49	**0.011**
Age groups (18–29 years)	6	1.00	0.50	0.076	0.67	0.50	0.363
(30–59 years)	35	0.89	0.77	**0.044**	0.77	0.57	**0.017**
(60+ years)	17	0.47	0.29	0.083	0.41	0.41	1.000
**Do you have health insurance?**							
Yes	11	0.82	0.64	0.167	0.82	0.55	0.082
No	51	0.76	0.59	**0.002**	0.61	0.49	0.083
**Do you have a chronic illness?**							
Yes	30	0.83	0.73	0.083	0.60	0.53	0.423
No	27	0.70	0.44	**0.006**	0.70	0.44	**0.017**
**Do you receive regular medical care for the treatment of any ongoing or chronic health problem?**							
Yes	47	0.89	0.74	**0.007**	0.68	0.57	0.096
No	14	0.43	0.14	**0.040**	0.57	0.21	**0.019**
**Do you receive regular medical care for the treatment of anxiety?**							
Yes	26	0.96	0.85	0.077	0.77	0.65	0.185
No	36	0.64	0.42	**0.000**	0.56	0.39	0.057
**Do you receive regular medical care for the treatment of depression?**							
Yes	22	1.00	0.91	0.083	0.91	0.77	0.083
No	40	0.65	0.43	**0.003**	0.50	0.35	0.083
**Do you have depression?**							
Yes	29	0.97	0.90	0.162	0.90	0.72	0.057
No	29	0.66	0.38	**0.002**	0.48	0.31	0.057
**Do you take medication daily?**							
Yes	48	0.83	0.67	**0.004**	0.69	0.54	**0.033**
No	12	0.67	0.42	0.082	0.58	0.42	0.339
**Screening for anxiety**							
Negative before and after	14	0.00	0.00	-	0.29	0.21	0.583
Improved after intervention	11	1.00	0.00	-	0.36	0.18	0.167
Positive before and after	37	1.00	1.00	-	0.86	0.70	0.057
**Screening for depression**							
Negative before and after	19	0.53	0.16	**0.005**	0.00	0.00	-
Improved after intervention	12	0.83	0.67	0.166	1.00	0.00	-
Positive before and after	28	0.93	0.86	0.161	1.00	1.00	-

Questions	Before and after	HAD-Anx (categorical % positive cases)			HAD-Dep (categorical % positive cases)		
		Before	After	Paired *t*-teste	Before	After	Paired *t*-teste
	(*n*)	Mean	Mean	*p*-value	Mean	Mean	*p*-value
**All patients**	62	**11.2**	**9.3**	**0.000**	**9.4**	**7.9**	**0.001**
**Gender** (Male)	9	9.8	7.6	**0.030**	8.4	7.8	0.591
(Female)	53	11.4	9.6	**0.001**	9.6	7.9	**0.001**
**Age groups** (18–29 years)	6	12.7	8.5	**0.042**	9.8	5.7	**0.003**
(30–59 years)	35	12.5	10.8	**0.008**	10.7	9.0	**0.010**
(60+ years)	17	8.1	7.0	0.144	7.1	6.8	0.790
**Do you have health insurance?**							
Yes	11	10.9	9.0	0.094	11.0	8.7	**0.032**
No	51	11.3	9.4	**0.000**	9.1	7.7	**0.009**
**Do you have a chronic illness?**							
Yes	30	12.3	11.3	0.088	9.1	8.8	0.561
No	27	9.6	7.1	**0.001**	9.7	6.8	**0.000**
**Do you receive regular medical care for the treatment of any ongoing or chronic health problem?**							
Yes	47	12.3	10.8	**0.003**	10.0	8.5	**0.006**
No	14	7.8	4.7	**0.008**	8.0	5.8	**0.009**
**Do you receive regular medical care for the treatment of anxiety?**							
Yes	26	13.2	11.9	0.077	11.0	9.2	**0.016**
No	36	9.8	7.4	**0.000**	8.3	7.0	**0.028**
**Do you receive regular medical care for the treatment of depression?**							
Yes	22	13.4	12.2	0.144	12.8	10.3	**0.002**
No	40	10.0	7.7	**0.000**	7.6	6.6	0.079
**Do you have depression?**							
Yes	29	13.4	11.5	**0.008**	12.5	10.1	**0.001**
No	29	9.8	7.9	**0.009**	7.3	6.3	0.094
**Do you take medication daily?**							
Yes	48	12.0	10.1	**0.000**	10.1	8.4	**0.002**
No	12	9.5	7.3	0.102	8.3	6.9	0.210
**Screening for anxiety**							
Negative before and after	14	4.4	4.1	0.583	5.4	5.8	0.649
Improved after intervention	11	11.0	5.2	**0.000**	6.8	3.9	**0.004**
Positive before and after	37	13.8	12.5	**0.020**	11.7	9.9	**0.005**
**Screening for depression**							
Negative before and after	19	7.9	5.3	0.007	4.2	3.8	0.439
Improved after intervention	12	11.7	9.1	0.068	10.4	5.6	**0.000**
Positive before and after	28	13.4	12.2	0.047	13.1	11.5	**0.020**

In red bold *p*-value < 5%.

The effect of the intervention also included a significant improvement in Quality of Life, mainly of the physical domain but also in the psychological and environmental ones. Quality of life is an important outcome in evaluating the effect of mindfulness practices as a health promotion intervention. Its greatest effect, as expected, was in increasing quality of life in the psychological domain, which was more significant among women and people under 60, with lower incomes, no chronic illnesses, and who did not receive regular medical care. In these groups, there is also an improvement in quality of life in physical terms. We noticed that the intervention is also significantly associated with improvement of quality of life in those with anxiety and depression, whether they were being treated or not, having a positive impact even on those who still have symptoms after the intervention. Interestingly, there was no such significant impact in the areas of social relationships and the environment. Only in those who do not face specific difficulties, i.e., have a better income, do not have a chronic illness and do not take medication regularly, improvements in the social relationships’ domain were found, associated with a reduction in the presence of anxious symptoms. This confirms another positive effect of these practices as forms of health promotion (Table 3).

**Table 3:** Evaluation of quality of life domains and screening for anxiety/depression, before and after intervention, in patients of the city of Rio de Janeiro/RJ, Brazil.

Questions	Before and after	Physical domain			Psychological domain		
		Before	After	Paired *t*-teste	Before	After	Paired *t*-teste
	(*n*)	Mean	Mean	*p*-value	Mean	Mean	*p*-value
All patients	62	**51.9**	**56.6**	**0.002**	**49.2**	**55.5**	**0.002**
Gender (Male)	9	56.3	63.9	0.066	61.1	63.9	0.572
(Female)	53	51.1	55.4	**0.013**	47.2	54.1	**0.002**
Age groups (18–29 years)	6	51.2	68.5	**0.028**	43.1	61.1	**0.010**
(30–59 years)	35	43.7	49.8	**0.003**	43.4	51.1	**0.004**
(60+ years)	17	67.8	65.3	0.238	60.0	59.9	0.965
**Do you have health insurance?**							
Yes	11	47.7	53.2	0.210	48.9	51.1	0.671
No	51	52.8	57.3	**0.006**	49.3	56.4	**0.001**
**Do you have a chronic illness?**							
Yes	30	48.8	50.9	0.284	49.8	50.9	0.697
No	27	57.1	64.2	**0.011**	50.5	60.6	**0.001**
**Do you receive regular medical care for the treatment of any ongoing or chronic health problem?**							
Yes	47	47.8	50.8	0.060	47.1	51.9	**0.039**
No	14	63.9	75.0	**0.009**	55.7	66.7	**0.014**
**Do you receive regular medical care for the treatment of anxiety?**							
Yes	26	42.7	48.7	**0.003**	42.9	51.7	**0.012**
No	36	58.5	62.3	0.095	53.7	58.3	0.069
**Do you receive regular medical care for the treatment of depression?**							
Yes	22	40.1	45.8	**0.013**	39.4	45.1	0.120
No	40	58.3	62.6	**0.044**	54.6	61.2	**0.008**
**Do you have depression?**							
Yes	29	39.5	46.3	**0.001**	37.3	47.1	**0.003**
No	29	61.1	65.0	0.119	57.9	62.2	0.087
**Do you take medication daily?**							
Yes	48	48.4	52.3	**0.013**	46.9	52.0	**0.042**
No	12	61.9	69.6	0.143	55.2	66.3	**0.000**
**Screening for anxiety**							
Negative before and after	14	74.1	75.3	0.381	68.2	69.3	0.696
Improved after intervention	11	61.0	71.8	**0.069**	59.8	69.7	0.137
Positive before and after	37	40.7	45.1	**0.025**	38.8	46.0	**0.006**
**Screening for depression**							
Negative before and after	19	65.6	70.9	0.107	66.9	70.6	0.291
Improved after intervention	12	49.9	57.7	0.091	46.2	61.5	**0.001**
Positive before and after	28	43.7	47.4	**0.050**	38.5	42.5	0.208

Questions	Before and after	Social relationships domain			Environment domain		
		Before	After	Paired *t*-teste	Before	After	Paired *t*-teste
	(*n*)	Mean	Mean	*p*-value	Mean	Mean	*p*-value
All patients	62	**52.8**	**57.4**	**0.048**	**46.5**	**48.3**	**0.240**
Gender (Male)	9	56.5	64.8	0.123	54.7	57.0	0.535
(Female)	53	52.2	56.1	0.126	45.1	46.9	0.313
Age groups (18–29 years)	6	51.4	58.3	0.474	40.1	46.4	0.152
(30–59 years)	35	48.3	52.9	0.169	43.9	45.9	0.351
(60+ years)	17	60.8	62.7	0.626	52.8	50.3	0.281
**Do you have health insurance?**							
Yes	11	45.5	55.3	**0.019**	54.8	53.7	0.739
No	51	54.4	57.8	0.199	44.7	47.2	0.159
**Do you have a chronic illness?**							
Yes	30	51.1	54.2	0.397	46.6	47.8	0.634
No	27	55.0	61.7	**0.029**	47.4	51.0	0.092
**Do you receive regular medical care for the treatment of any ongoing or chronic health problem?**							
Yes	47	51.2	55.0	0.190	44.1	45.6	0.408
No	14	56.6	64.9	**0.025**	53.9	56.3	0.407
**Do you receive regular medical care for the treatment of anxiety?**							
Yes	26	46.7	51.9	0.220	44.9	49.1	0.085
No	36	57.2	61.3	0.119	47.7	47.8	0.960
**Do you receive regular medical care for the treatment of depression?**							
Yes	22	45.0	47.0	0.650	42.1	43.6	0.495
No	40	57.1	63.1	0.028	49.0	50.9	0.345
**Do you have depression?**							
Yes	29	44.5	49.1	0.203	40.7	43.9	0.141
No	29	59.5	64.1	0.175	50.2	51.6	0.555
**Do you take medication daily?**							
Yes	48	51.5	54.3	0.304	44.1	45.8	0.371
No	12	56.3	68.1	**0.016**	53.9	56.9	0.261
**Screening for anxiety**							
Negative before and after	14	65.5	70.2	0.140	59.6	59.0	0.780
Improved after intervention	11	58.3	69.7	**0.050**	46.9	54.5	0.088
Positive before and after	37	46.4	48.9	0.446	41.5	42.5	0.633
**Screening for depression**							
Negative before and after	19	64.1	71.0	0.062	54.9	56.1	0.620
Improved after intervention	12	51.4	59.7	0.119	42.6	51.7	**0.028**
Positive before and after	28	45.2	47.6	0.517	42.8	41.5	0.587

In red bold *p*-value < 5%.

### 3.2 Qualitative results

The following thematic categories emerged from the qualitative analysis of the focus groups: access to mindfulness groups and motivations for seeking this type of intervention, opportunity to experience mindfulness, daily life experiences that influence adherence, coping strategies for difficulties and challenges, benefits of the mindfulness group on emotional suffering, changes in perception about mindfulness and meditation. There were also themes related to the benefits of participating in groups in general, as well as changes in self-perception. These themes are discussed below.

#### 3.2.1 Access to the groups - motivations and references

When the qualitative study is considered, most users reported joining the group because of referrals from the unit’s professionals, generally related to stress management, anxiety, or depression symptoms, or even support for people with chronic pain. Exceptions were people who join the group based on referrals from other family members, friends, and neighbors. The engagement of patients in the groups fundamentally involves pre-established bonding and trust with the FHT.

I’m 53 years old. I came because the physiotherapist mentioned that there was going to be a meditation group and I was curious. I’ve always really enjoyed learning, for me too as I’m now entering menopause, I have insomnia, and a lot of headaches.

#### 3.2.2 A long-awaited opportunity

Mindfulness groups in SUS also caters for a portion of the population who are curious and want to try it out but cannot afford private groups in which participation fees are charged. Some statements show the gap in public policies and the importance of the availability of free secular mindfulness meditation spaces. Some users reported looking for mindfulness courses or groups and finding them unaffordable. This shows that, despite its popularity and the existence of a large number of courses, workshops, teaching materials and apps, mindfulness is still inaccessible to a significant portion of the Brazilian population, especially those who use the SUS as their main source of care.

I thought I had to pay, because I’ve always been interested in other meditation courses, but I couldn’t afford them. So, I’ve always been interested, and knowing that we now have a meditation group at the family health unit was good.

#### 3.2.3 Family stress, family solutions

Analysis of the focus groups showed that several patients who took part in the mindfulness groups are caregivers of family members with chronic illnesses or disabilities. The motivation to take part in the mindfulness groups came from their perception of the emotional suffering related to tending to their loved ones. Among these patients involved in the daily care of their families, the strategy was to incorporate the family as co-participants in the mindfulness practices carried out at home. At their homes, where people often have little or no privacy, if they are to have a brief pause to practice mindfulness, it needs to be shared, so that it makes sense to their peers.

My home it's a problem for me, until I get out of there, I don't think I'll ever get better. And my family says that what I have is spiritual, but I'm completely sure that it's not spiritual. Then, from that day on, I started doing various (mindfulness) exercises, and I was able to take more control of the situation, which I didn't have before. I started to do the exercises and got my mother to do them. My mother wouldn't let me be quiet at home, either. I'd say Mom, look, I'm doing a meditation that goes like this, like that, right? Then I started explaining it to her. I got the CD and now I do it with her, at home. Now even my brother is doing it with us…

#### 3.2.4 Time for myself

Another aspect valued by the participants was the possibility of having quality time to themselves. Both the time set aside to take part in the weekly group, but also the time set aside to practice at home as a moment of self-care.

The most important thing is that I've discovered, in this fast-paced life, I've learned to find time for myself. (.)This solves a lot of problems, finding time for yourself in the midst of all the hustle and bustle solves a lot of problems. When you come here to the clinic, you get out of your day-to-day life for a special day. In everyday life, practicing is different, but I was able to do it at home too.

#### 3.2.5 Focus, attention and wellbeing

The patients also reported that the effects of the mindfulness groups involved improving focus and attention, greater body awareness, greater awareness of thoughts and emotional reactions. From these experiences, they observed an improvement in emotional regulation and a reduction in symptoms of anxiety, insomnia, and depression.

I realized that I'm still anxious, but with more inner space. I'm managing to control myself, which I used to not be able to do. Now I don't, I know when it's going to start, I can divert my thoughts, you know? I look after my mother, so I used to get very nervous, but I'm more patient with her, you know? Now I feel I have more patience. I'm managing to control my anxiety.

One thing that improved a lot was insomnia. I used to have a lot of insomnia. So, even if I wake up at night, I can meditate and I can go back to sleep. And that was great because I'm extremely anxious. Sleepless nights are horrible.

Increased body awareness changed some patients’ experience of daily life, through habits such as walking and eating. The practices helped them not only to cope with unpleasant experiences but also to enjoy pleasant moments.

Dancing I could clearly feel the awareness of my movements. That's what I've felt the most, because I've improved in ballroom dancing because of the practices here. It's been very good for me. I'm very anxious. I knew how to do the step, but when it came time to do it, I wanted to run over my partner. And I'm trying to reconcile all this with my breathing, you know.

The practice of eating raisins with full attention was one that benefited me. Because of my anxiety, I eat too fast, and the technique helped me to savor the food. I started eating more slowly and I no longer feel sick to my stomach.

Throughout the intervention, participants observed changes in attitudes related to self-perception, regarding feelings, emotions and thoughts. It was possible to increase kindness towards oneself and reduce reactivity to the thoughts content in the sense of emotional self-regulation. These skills proved useful in dealing with anxiety symptoms and unpredictable or undesirable everyday events.

The instructors always said: during the practices, we should be very gentle with ourselves. That was something I also took into my life, so, when I'm doing something, if it doesn't turn out the way I wanted, I should be gentle with myself.

#### 3.2.6 Another form of care

Many patients recognize group psychosocial interventions strategies as a coherent offer in PHC, complementary to individual and medication treatments. In the sample studied, mindfulness groups emerged as a possibility for managing anxiety with the expectation of reduction and even withdrawal of anxiolytic medication.

Some of the patients’ motivation was associated with dissatisfaction with the usual treatments offered, such as anxiolytic medication and other types of group psychotherapy.

In group therapy you have to expose your life in front of everyone, or sit in front of a psychologist and all you have to do is talk and he'll say “keep taking the medication,” the same thing with the psychiatrist. And not here, here is a group where little by little we become aware that it's not like that, and over time maybe we'll reduce the medication, because I take very strong medication for depression.

It’s interesting to note that the presence of mindfulness groups as an ordinary PHC offering can set up a space in which some patients can negotiate other forms of mental health care, either as a substitute or as a complement to their usual treatment. When asked about the possible impacts of the group, in terms of benefits and difficulties, participants reported changes in their relationship with painful symptoms and the experience of illness.

Well, I was pretty unwell when I came here, but along with these practices I went to the doctor. I took some pills that he gave me, it was only one box. He even said “Are you better?” And I said, “I am”. And I did get better. Then, combining the group here with the medication, I got better.

#### 3.2.7 Benefits of being in a group

The patients recognize that the benefits of the group go beyond the skills developed over the 8 weeks; they also involve socialization, networking, and mutual help.

Because for me at least, the group is welcoming. It's not just the practices themselves. Understanding that there are other people in the same situation. And that we can help each other. And that these practices help us.

#### 3.2.8 Difficulties

Some participants reported difficulties in the group related to the infrastructure of the health units and the ambience.

I think one thing that had a big influence on my difficulty was the atmosphere in the clinic: every time someone knocked on the door, someone opened the door, there was a lot of noise, a lot of people talking. The accommodation itself, the benches, the low chair, the discomfort didn't allow us to relax enough to enjoy it. That was my difficulty.

#### 3.2.9 Surprises and adjustments - mindfulness and patients’ religious context

One of the concerns about offering mindfulness groups in the context of PHC would be the possibility of reconciling secular practices with the patients’ religious context.

The participants indicated that before starting the groups, they thought that mindfulness might have a religious component, but this was not sustained when they actually attended the sessions. Some of the patients even incorporated mindfulness practices into their current religious practices in church.

I thought it was church stuff until I did the meditation.

I even practice mindfulness at church. Breathing brought changes, I felt more confident.

The highlighted excerpts point out that the construction of meaning for participation is anchored in the experience of mental suffering and in the opportunities offered by the PHC teams for their patients. Mindfulness groups then emerge as an option for relieving suffering, even if the mechanisms involved in the treatment process are not debated or made explicit to participants by professionals.

## 4 Discussion

### 4.1 Key findings

This original quali-quanti study in the Brazilian population confirmed that mindfulness-based groups are effective as a psychosocial intervention for health promotion and to improve quality of life and mental health care. The findings of our research are compatible with review studies in primary care, which have also observed positive impacts on quality of life and mental health with mindfulness-based interventions (29). They can be held by professionals from the PHC teams and specialized professionals as part of collaborative care. The benefits of participating in the groups for the management of these complaints appeared both in the quantitative results and in the perception of the participants in the focus group reports. The patients demonstrated a significant increase in Mindfulness and Self Compassion, associated with reduction of anxiety and depression symptoms and increase of Quality of Life. Patients’ motivations for taking part in the group ranged from curiosity about meditation, a desire to manage the stress of daily life, and control of symptoms such as insomnia, anxiety, and depression. The patients pointed out that the practices were helpful in self-regulating emotions, feelings, and bodily sensations. They also described the ways they assimilate the practices they had experienced at the health unit in their daily lives, adjusting and negotiating their use in new scenarios. As the practices were recommended by health professionals, they assumed a status of being a therapeutic intervention. Thus, it can be considered as a complementary intervention that will help patients develop self-esteem and empowerment, increasing wellbeing. Our study reinforces that mindfulness-based interventions can be successfully used in PHC in the National Health System in Brazil.

The study also points to the fact that a proportion of people suffering from mental health conditions find themselves without regular follow-up and treatment, even if they are regularly attending primary care, indicating a gap in mental health care, something that has been observed in other research related to mental health (2, 30). Considering the great variability of people being treated in primary care services, being able to offer different therapeutic strategies can improve access and equity for groups with different needs. Recognizing the mental health care gap and the presence of psychological distress and mental health symptoms prior to a mindfulness-based program helps to recognize the groups that can most benefit from this type of intervention. A meta-analysis investigating different interventions that can improve mental wellbeing showed that mindfulness benefits among the general population and in clinical settings. Another meta-analysis of mindfulness programs and health promotion showed that individual characteristics related to worse mental health outcomes seem to be related to better outcomes, with a greater influence than gender and educational level (31).

Our research also indicates that it is possible to offer 8-week mindfulness groups in PHC with the involvement of health teams. Primary care services are a favorable setting for stepped care of different intensities and durations (32), but a randomized study carried out with PHC patients shows that an eight-week structured intervention has better results than brief interventions in terms of self-regulation and self-care for this clientele (33). Thus, our research helps to reinforce that, if qualified instructors are available to offer protocols adapted for PHC, it is possible to provide an intervention with real benefits for the population in question.

Although there exist few specific studies on mindfulness interventions with disadvantaged Brazilian population, there are some experiences with people in situations of vulnerability, such as immigrants, which have similar results to this research. A study carried out with Portuguese-speaking immigrants in Boston showed that a mindfulness program adapted for PHC was acceptable, feasible and culturally appropriate for this population (34). Studies with Latino/Hispanic populations show that adapted protocols have greater adherence, and significant results in reducing depressive symptoms, stress and symptoms related to chronic diseases (35, 36). Other studies carried out with overweight low-income Brazilian women show that mindfulness-based interventions can help cultivate perceived self-compassion and positively affect the participants’ lives (37).

Studies in Brazilian primary care show that women are the main users of primary care services, including participating in psychosocial interventions (1). It is therefore not surprising that most of the sample in our study was made up of women. Although there is no research on mindfulness and meditation use profiles with the Brazilian population, the findings of this study are compatible with US population studies in which most people engaging in mind–body meditation practices are female (38). The manifestation of emotional distress is more frequent in women, which in our study appears to be due to the stress of being a caregiver and not having time for oneself. Mindfulness groups could be a coping strategy for these women. Qualitative studies with women in situations of extreme vulnerability in the role of family caregiver show that the quality time for oneself and emotional self-regulation promoted by mindfulness-based interventions can improve quality of life and interpersonal relationships (39). The perceptions of the participants in our research are also compatible with reviews that indicate that mindfulness-based interventions can help informal caregivers of people with chronic illnesses (40, 41).

We did not expect to find practices from a different spiritual tradition (even if it is secularized to a certain extent, as mindfulness activities are) being incorporated so easily and without conflict into users’ religious practices (mostly Catholic or Neo Pentecostal) in very poor communities. This ease may have happened because Brazilian syncretism facilitates the translation of elements from a particular religious practice into another (there are many examples: Afro-Brazilian religions such as Umbanda which incorporate elements from the Catholic tradition). Brazilian culture has an enormous capacity for including elements from other cultural traditions, and this explains the acceptance of mindfulness, which may be different in other contexts (42, 43). This finding is very promising concerning how cultural differences can be integrated within mindfulness practices.

These qualitative research results are aligned with the most recent reflections in the field of mindfulness on the need to build intercultural and interreligious competencies in order to strengthen the presence of mindfulness-based interventions as a public health care option (44). Mental health interventions must respect the patients’ beliefs and values system while helping to maintain the integrity of the professionals and the fidelity of the proposed intervention (45). Mindfulness-based interventions are presented in the Global Mental Health literature as a successful example of integrating and adapting a health care strategy from a traditional system (46). Mindfulness-based programs can currently be found in more than 50 countries across the world. But there is little research into cultural influences and potential adaptations, especially in Latin-American countries, and our study did not show special resistance from the patients while highlighting the importance of professionals responsible for PHC recommending them (47, 48).

Implementation studies show that health professionals recommend therapies and treatments that they recognize as coherent and effective in their work setting (32). Bringing information and experiences about mindfulness to PHC professionals could be a way of increasing the availability of the intervention for patients. Previous research carried out in Brazilian PHC with health professionals indicates that mindfulness-based interventions are feasible (49). The research findings with Brazilian health workers are compatible with studies carried out in other countries with PHC professionals who work with vulnerable populations. The continuous offer of groups with the possibility of maintenance sessions, combined with continuity of care and the ongoing therapeutic bonds with health teams, are relevant to promote and implementing behavior activation, including changing habits and increasing self-care, in PHC.

It is important to keep on investigating how to incorporate mindfulness practices in universal health systems as the Brazilian SUS. We can point that an important aspect to be investigated involves the minimum amount of time for an intervention to last. Moreover, it is fundamental to evaluate the possibilities of developing opportunities for continuing the practices. The participants have talked about the difficulties of maintaining them within their daily routine in small and crowded homes. Including mindfulness practices in other health promotion group activities that are held at the primary care units have shown to be feasible and easily accepted. Keeping open and public mindfulness groups as a form of follow-up after the end of the protocols, even though mobile messaging apps proved to be a strategy recognized by professionals and patients as effective, but they need to be better implemented and evaluated.

### 4.2 Potential shortcomings and limitations

The paper offers novel insights into implementing mindfulness-based interventions in resource-limited settings, which is a significant contribution to the field. A special aspect of this project is that it has trained Brazilian professionals from the National Health System to use a protocol specially designed for our Health system and population – The MBHP – Mindfulness Based Health Promotion (50) which limits its scalability. On the other hand, a main limitation of our study is the absence of follow-up for a longer period. Medium and long-term follow-up studies, participant observation of groups and other methodologies for mixed methods could contribute to a more comprehensive understanding of mindfulness-based interventions as an alternative for stress management, mental health, and well-being promotion in patients in primary health care in LAMIC. It would also have been important to analyze why patients dropped out, even these being in frequent group activities offered in PHC. A better understanding of reasons could provide new insights for both mental health services and the implementation of mindfulness groups.

Another limitation of our study is the potential for selection bias, as participants who volunteered for a mindfulness-based intervention might already have been more open to or in need of psychological support. This selection bias could limit the generalizability of our findings to the wider population of primary care patients. Deepening our knowledge of the demographic characteristics of group participants in terms of educational level, ethno-racial characteristics, and patterns of use of health services can also provide new insights into social markers of difference that can influence the demand for and potential benefits of groups for the health of the population. The fact that less than 50% of the patients participating in the groups answered the post-intervention tests demonstrates the difficulty of the analyzes of “quasi experimental studies”.

Moreover, while our findings are promising, they should be interpreted with caution due to the study’s limited sample size and the absence of a control group. Future studies could address these limitations by including a larger, more diverse sample and employing a randomized controlled trial design to provide more robust evidence of the intervention’s effectiveness.

Finally, the generalizability of our results to other Low and Middle-Income Countries (LAMICs) may be limited, given the unique sociocultural context of Rio de Janeiro. Subsequent research in varied geographical and cultural settings would be valuable to ascertain the wider applicability of our findings.

## 5 Conclusion

The results of the research indicate that MBHP groups are a viable option for psychosocial intervention in the primary health care scenario in Brazil’s. They have shown to be a feasible, well-accepted and efficacious method of offering support and promoting well-being for a low-income population in PHC. Observations of both the patients’ impressions and the results of the scales applied in the pre-and post-intervention period indicate that the groups carried out using the MBHP protocol had an effect on different spheres of the health of the population studied, both in terms of mental health promotion, quality of life increase, and the management of physical symptoms and anxiety. The findings of our study are particularly relevant for healthcare practitioners and policymakers in LAMICs, offering valuable insights into the implementation of patient-centered, mindfulness-based interventions in resource-limited settings. By demonstrating the feasibility and benefits of such interventions, this research contributes to the ongoing efforts to empower patients and foster a more patient-centered approach in global health care, particularly within vulnerable communities.

## Data availability statement

The raw data supporting the conclusions of this article will be made available by the authors, without undue reservation.

## Ethics statement

The studies involving humans were approved by Approved by the Research Ethics Committees of State University of Rio de Janeiro (UERJ) and the Health Department of Rio de Janeiro Municipality. CAAE: 39405814.3.0000.5259. The studies were conducted in accordance with the local legislation and institutional requirements. The participants provided their written informed consent to participate in this study.

## Author contributions

DS: Conceptualization, Formal analysis, Methodology, Project administration, Writing – original draft, Writing – review & editing. SF: Formal analysis, Methodology, Project administration, Writing – original draft, Writing – review & editing. CK: Funding acquisition, Project administration, Writing – original draft. KA: Project administration, Writing – original draft. MC: Conceptualization, Formal analysis, Methodology, Writing – review & editing. AN: Writing – review & editing. FO: Methodology, Writing – review & editing. JG-C: Conceptualization, Formal analysis, Methodology, Writing – original draft. MD: Conceptualization, Formal analysis, Methodology, Project administration, Writing – review & editing.
